# Ovicidal and repellent activities of several plant essential oils against *Periplaneta americana* L. and enhanced activities from their combined formulation

**DOI:** 10.1038/s41598-022-16386-x

**Published:** 2022-07-15

**Authors:** Mayura Soonwera, Tanapoom Moungthipmalai, Wacharaporn Takawirapat, Sirawut Sittichok

**Affiliations:** Department of Plant Production Technology, Faculty of Agricultural Technology, King Mongkut’s Institute of Technology Ladkrabang, Ladkrabang, 10520 Bangkok Thailand

**Keywords:** Entomology, Plant sciences

## Abstract

Natural ovicidal and repellent agents against *Periplaneta americana* L. are urgently needed, and plant essential oils (EOs) can assume this role quite readily. In this study, ovicidal and repellent activities against *Periplaneta americana* of EOs from *Cymbopogon citratus* (Stapf.), *Cinnamomum verum* (J. Presl.), *Eucalyptus globulus* (Labill.), *Illicium verum* (Hook.f.), and *Zanthoxylum limonella* (Alston) in soybean oil and in ethyl alcohol were determined by topical and dual-choice assays, as well as 10% cypermethrin and a combined formulation of 5% *C*. *verum* EO + 5% *I*. *verum* EO. Cypermethrin at 10% provided the highest toxicity (100% inhibition rate) against the eggs, but only slightly higher than that (99.3%) provided by the combined EO formulation, while the highest repellent activity against the adults was provided by the combined formulation (89.5% repelled cockroaches at 48 h after treatment). In addition, all EO formulations in soybean oil provided higher ovicidal and repellent activities than those in ethyl alcohol. To conclude, the combined EO formulation in soybean oil can replace cypermethrin because their efficacy was nearly equivalent, but the combination should be much safer to use.

## Introduction

Cockroaches are unhygienic scavenger insect pests in human settlements^1^. They belong to the Order Blattodea^2^. Only 25–30 out of 4500–4600 cockroach species are associated with human habitation and classified as pests^2,3^. American cockroach, *Periplaneta americana* L. (*P*. *americana*) (Blattidae), is one of the most serious pests in houses, buildings, farms, and livestock areas^3,4^. The color of this insect pest can be reddish brown to brownish black, with a body length of 34–53 mm. Their body shape is a flattened oval shape. The body of a male adult is longer than that of a female adult^3,4^. Female adults deposit one ootheca (egg case) per month for at most ten months. Each ootheca contains 14–16 eggs^3,4^. *P. americana* undergoes incomplete metamorphosis in its life cycle. Its three life stages of eggs, nymphs, and adults can extend from 168 to more than 700 days^3,4^. Eggs may take between 30 and 60 days to hatch into nymphs. Nymphs grow into adults after 6 to 12 months. The adult life span was 1–12 months, and a female cockroach can reproduce a total of 100–150 young nymphs during her lifetime^3,4^. The length of each of its life stages, as well as the length of its lifetime, are influenced by temperature, humidity, and amount of available food^3,4^. It is a nuisance pest that causes psychological distress and public health problems because it mechanically transmits over 100 types of human pathogenic organisms, foodborne pathogens, and parasites, such as *Citrobacter freundii*, *Escherichia coli*, *Enterococcus faecalis*, *Pseudomonas aeruginosa*, *Proteus vulgaris*, *Staphylococcus aureus*, *S*. *epidermidis*, *Aspergillus niger*, *Saccharomyces cerevisiae*, *Ascaris lumbricoides*, *Balantidium coli*, *Enterobius vermicularis*, *Entamoeba histolytica*, *Schistosoma haematobium*, and *Trichuris trichiura*^5–8^. Today, *P*. *americana* is everywhere around the globe and is spread throughout it by global commerce^5,6^. Long-term control of *P*. *americana* is complicated because it is highly adaptable. It can feed not only on all human foods but also on many other organic objects that are not human foods (book binding, cardboard, fresh & dried blood, excrement, sputum, baby fingernail, dead & crippled cockroaches)^3,4^. At the time of the study, it was resistant to most conventional insecticides, such as malathion, carbaryl, permethrin, and cypermethrin^9,10^. An accepted strategy for current cockroach control is to reduce the use of synthetic insecticides^11^. The ultimate goal of this research project was to find a safer and at least as effective alternative to these synthetic insecticides because they (e.g., organophosphates and carbamates) are deadly poisonous to humans^12,13^.

Many researchers have suggested using plant extracts and essential oils (EOs) as good alternatives^14–17^ to synthetic insecticides. There have been a large number of reports that some EOs exhibited strong repellent activity against *P. americana* adults: *Boesenbergia rotunda*, *Citrus hysteric*, *Cymbopogon citratus*, *C*. *nardus*, *Curcuma longa*, *Eucalyptus citriodora*, *Jatropha curcas*, *Litsea cubeba*, *Mentha arvensis*, *Piper nigrum*, *Psidium guajava*, *Syzygium aromaticum*, and *Zingiber* officinale^18–21^. In addition, a combination of *E*. *globulus* + *Rosmarinus officinalis* EOs showed a stronger insecticidal effect against adult *P*. *americana* than that of each individual EO^16^. Finally, plant EOs are much safer and more environmentally friendly than conventional synthetic insecticides^10,14^. Plant EOs from *C*. *citratus*, *Cinnamomum verum*, *E*. *globulus*, *Illicium verum*, *Pimenta dioica*, *Myristica fragrans*, and *Zanthoxylum limonella* have been reported before to possess insecticidal activities, to be safe for human, used safely since ancient times, as spices in Thai and Asian foods, and as antibiotics in folk medicine^22–30^.

Therefore, the first objective of this work was to determine the ovicidal and repellent activities of EOs extracted from the following plant parts: *C*. *citratus* stems, *Cinnamomum verum* (*C*. *verum*) bark, *Eucalyptus globulus* (*E*. *globulus*) leaves, *Illicium verum* (*I*. *verum*) fruits, and *Zanthoxylum limonella* (*Z*. *limonella*) fruits*.* The second objective was to combine the EO that provided the highest ovicidal activity with the EO that provided the highest repellent activity into a strong formulation against the eggs and adults of the American cockroach, *P*. *americana*, and determine the formulation’s potency. If the combined formulation was demonstrated to be equivalently effective to cypermethrin, it can be developed into a safer ovicidal and repellent agent for comprehensive control of cockroach populations in epidemic areas, which can help eliminate human pathogenic organisms transmitted by *P*. *americana*.

## Results

Essential oils (EOs) of*C*. *citratus* stems, *C. verum* bark, *E*. *globulus* leaves, *I*. *verum* fruits, and *Z*. *limonella* fruits were extracted by a water distillation method, and their chemical constituents were identified by GC-MS. The identified constituents and the percentage yield of each EO are listed in Table 1. The EO with the highest percentage extraction yield was *Z*. *limonella* EO (9.27% v/w), followed by *I*. *verum* EO (3.23% v/w), *E*. *globulus* EO (1.21% v/w), *C. citratus* EO (1.15% v/w), and *C*. *verum* EO (1.10% v/w). The *C*. *citratus* EO was composed of 10 chemical constituents, accounting for 97.40% of the chemical profile. Its major constituents were geranial (46.33%), neral (25.72%), and 1-8-cineole (10.93%). The *C*. *verum* EO was composed of 11 chemical constituents, accounting for 98.60% of the chemical profile. Its major constituents were cinnamaldehyde (75.23%) and benzene (14.15%). *E*. *globulus* EO was composed of 19 chemical constituents, accounting for 97.35% of the chemical profile. Its major constituents were 1,8-cineole (45.82%) and ϒ-terpinene (20.13%). The *I. verum* EO was composed of 10 chemical constituents, accounting for 98.58% of the chemical profile. Its major constituent was trans-anethole (92.24%). *Z*. *limonella* EO was composed of 16 chemical constituents, accounting for 97.75% of the chemical profile. Its major constituents were limonene (28.13%) and terpinen-4-ol (24.13%).

**Table 1:** Chemical constituents of *Cymbopogon citratus*, *Cinnamomum verum*, *Eucalyptus globulus*, *Illicium verum*, and *Zanthoxylum limonella* and their percentage yield in the total chemical profile. ^a^R = Retention index of a chemical constituent determined with an HP-5 MS column and compared with the retention indices of standard alkanes (C_7_-C_30_) for identity verification. ^b^K = Kovat retention index from NIST17^62^. ^c^I = Identified and confirmed by mass spectrum (M) matching with chemicals in the computer mass library of Adams^61^ and by retention index (R) matching with those reported in NIST17^62^.

Item	Compound	R^a^	K^b^	Peak area (%), (average of 3 runs ± SD)					I^c^
				*C*. *citratus*	*C*. *verum*	*E*. *globulus*	*I*. *verum*	*Z*. *limonella*	
1.	Acetoin	680	680	–	–	–	0.41 ± 0.02	–	M,R
2.	α-Thujene	928	930	–	–	–	0.21 ± 0.01	2.31 ± 0.54	M,R
3.	α-Pinene	932	933	3.52 ± 0.11	0.91 ± 0.02	5.01 ± 0.03	0.32 ± 0.02	1.83 ± 0.08	M,R
4.	Camphene	946	946	–	0.52 ± 0.01	0.32 ± 0.02	–	–	M,R
5.	Sabinene	977	977	–	–	3.75 ± 0.06	–	4.52 ± 0.05	M,R
6.	Β-Mycene	990	991	–	0.43 ± 0.03	0.31 ± 0.01	–	0.32 ± 0.02	M,R
7.	α-Phellandrene	1002	1003	–	0.32 ± 0.01	1.67 ± 0.10	–	1.31 ± 0.07	M,R
8.	α-Terpinene	1002	1002	0.22 ± 0.08	–	–	0.31 ± 0.01	4.82 ± 0.18	M,R
9.	Benzene	1011	1009	–	14.15 ± 0.25	8.32 ± 0.38	–	8.21 ± 0.76	M,R
10.	1,8-Cineole	1024	1025	10.93 ± 1.01	0.61 ± 0.02	45.82 ± 1.01	0.52 ± 0.03	–	M,R
11.	Limonene	1029	1029	–	–	–	1.85 ± 0.09	28.13 ± 1.37	M,R
12.	ϒ-Terpinene	1051	1050	0.26 ± 0.14	–	20.13 ± 1.44	–	7.82 ± 0.82	M,R
13.	Butanoic acid	1085	1086	–	–	0.52 ± 0.06	–	–	M,R
14.	Linalool	1086	1086	0.86 ± 0.07	–	–	–	1.15 ± 0.07	M,R
15.	Terpinolene	1089	1088	–	–	1.12 ± 0.61	0.23 ± 0.01	2.23 ± 0.83	M,R
16.	D-Fenchyl alcohol	1110	1110	–	–	0.55 ± 0.04	–	–	M,R
17.	*trans-*Pinocaveol	1139	1139	–	–	0.72 ± 0.05	–	–	M,R
18.	Borneol	1146	1147	–	1.23 ± 0.08	0.81 ± 0.01	–	–	M,R
19.	Terpinen-4-ol	1165	1166	–	–	3.21 ± 0.23	–	24.13 ± 1.65	M,R
20.	*trans*-Carveol	1204	1204	–	–	0.23 ± 0.01	–	1.73 ± 0.82	M,R
21.	Myrtenol	1214	1214	–	–	0.71 ± 0.02	–	–	M,R
22.	Neral	1217	1217	25.72 ± 1.97	–	–	–	–	M,R
23.	Cinnamaldehyde	1222	1222	–	75.23 ± 2.17	–	–	–	M,R
24.	*p-*Anisaldehyde	1223	1223	–	–	–	1.63 ± 0.02	–	M,R
25.	Carvone	1230	1231	–	–	–	–	2.47 ± 0.08	M,R
26.	Geraniol	1237	1237	5.71 ± 0.82	–	–	–	–	M,R
27	Geranial	1247	1247	46.33 ± 1.32	–	–	–	–	M,R
28.	α-Terpineol	1280	1279	–	–	2.85 ± 0.49	–	6.23 ± 1.12	M,R
29.	*trans-*Anethole	1283	1285	–	–	–	92.24 ± 1.83	–	M,R
30.	Eugenol	1356	1355	–	–	–	0.84 ± 0.03	–	M,R
31.	Geranyl acetate	1379	1380	3.87 ± 0.33	–	–	–	–	M,R
32.	Copaene	1380	1381	–	2.02 ± 0.15	–	–	–	M,R
33.	Cinnamic acid	1460	1462	–	0.33 ± 0.09	–	–	–	M,R
34.	α-Gurjunene	1527	1529	–	–	0.86 ± 0.02	–	–	M,R
35.	Epiglobulol	1563	1564	–	–	0.44 ± 0.01	–	–	M,R
36.	Caryophyllene	1578	1580	1.17 ± 0.87	–	–	–	0.54 ± 0.02	M,R
37.	Cadalene	1659	1659	–	0.32 ± 0.08	–	–	–	M,R
	Total (%)			97.40	98.60	97.35	98.56	97.75	–
	Yield (%)			1.15	1.10	1.21	3.23	9.27	–

The inhibition rates of every treatment and control against *P*. *americana* eggs at 30 days after exposure are tabulated in Table 2. All treatments and controls were 10% solutions of a compound in soybean oil and in ethyl alcohol. The inhibition rate of every EO formulation in soybean oil (85.3–96.7%) was higher than that of the same EO in ethyl alcohol (60.3–90.1%). The highest egg inhibition rate, at 96.9%, among all 10% EO solutions in soybean oil, was exhibited by *I*. *verum* EO, and the lowest egg inhibition rate, at 85.3%, was shown by *C*. *verum* EO. The highest inhibition rate among all 10% EO formulations in ethyl alcohol, at 90.1%, was achieved by *Z*. *limonella* EO, and the lowest, at 60.3%, was exhibited by *C*. *citratus* EO*.* The implications of these highest and lowest inhibition rate results are separately discussed in the discussion section. In contrast, 10% w/v cypermethrin both in soybean oil and in ethyl alcohol exhibited a full 100% inhibition rate, the highest among all treatments. Regarding the inhibition rate index (IRI), every EO formulation showed an IRI of less than 1 (0.6-0.97), i.e., they were less toxic against *P*. *americana* eggs than 10% w/v cypermethrin.

**Table 2:** Inhibition rate at 30 days against *Periplaneta americana* eggs of 10% solutions of five plant essential oils (EOs) and cypermethrin in soybean oil and in ethyl alcohol. *IRI* Inhibition Rate Index, *S* Soybean oil solution, *E* Ethyl alcohol solution, ^*^Treatment and non-treatment are significantly different at *P* *<* 0.05.

Treatment	Solvent	Number of hatched eggs ± SD		Inhibition rate (%)	*P*-value	IRI
		Treated	Untreated			
*C*. *citratus* EO	S	12.8 ± 1.9^*^	145.1 ± 5.3	91.2	0.03	0.91
	E	55.7 ± 4.8	140.3 ± 6.4	60.3	0.08	0.60
*C*. *verum* EO	S	20.3 ± 3.4^*^	138.2 ± 6.7	85.3	0.04	0.85
	E	27.5 ± 4.8^*^	140.5 ± 7.4	80.4	0.10	0.80
*E*. *globulus* EO	S	16.2 ± 2.7^*^	128.7 ± 5.9	87.4	0.03	0.87
	E	40.7 ± 4.8	130.8 ± 5.3	68.9	0.10	0.69
*I*. *verum* EO	S	3.9 ± 1.8^*^	125.7 ± 2.8	96.9	0.01	0.97
	E	18.8 ± 2.7^*^	130.7 ± 3.9	85.6	0.06	0.86
*Z*. *limonella* EO	S	9.6 ± 1.9^*^	132.4 ± 5.5	92.7	0.01	0.93
	E	23.7 ± 4.3^*^	137.2 ± 4.8	82.7	0.05	0.83
Cypermethrin	S	0^*^	148.4 ± 3.7	100	0.01	–
	E	0^*^	152.3 ± 6.8	100	0.01	–

The percentage repellent rates against *P*. *americana* adults at 48 h of exposure of treatments and control in soybean oil and in ethyl alcohol are shown in Table 3. All 10% solutions in soybean oil of the five plant EOs exhibited a high percentage range of repellent activity, of 60.8-100%, against *P*. *americana* adults, and each of them exhibited a higher percentage repellent activity than the same EO in ethyl alcohol (47.6–88.2%).

**Table 3:** Repellent activities against *Periplaneta americana* adults of 10% solutions of five plant essential oils (EOs) and cypermethrin in soybean oil and in ethyl alcohol. Mean percentage repellent activity within the same column followed by different letters are significantly different at *P<*0.05 (ANOVA and Tukey’s post hoc test). *S* Soybean oil formulation, *E* Ethyl alcohol formulation, *R*^*2*^Regression coefficient, ^*^Significantly different at *P* *<* 0.05.

Treatment	Solvent	% Repellent / Time (h)					Regression equation of time versus % repellent	*R*^2^
		1	6	12	24	48		
*C*. *citratus* EO	S	98.0 ± 4.0^a^	93.8 ± 5.1^a^	92.8 ± 6.1^a^	92.0 ± 6.8^a^	79.0 ± 1.9^a^	y= −0.1228x+93.257	0.94
	E	88.2 ± 5.9^b^	78.4 ± 3.6^b^	78.0 ± 2.4^b^	69.6 ± 1.8^b^	62.0 ± 2.4^b^	y= −0.1735x+92.192	0.92
*C*. *verum* EO	S	100^a^	91.0 ± 3.7^a^	88.6 ± 1.9^a^	88.4 ± 1.7^a^	83.4 ± 3.4^a^	y= −0.0967x+93.764	0.94
	E	84.8 ± 4.5^b^	80.2 ± 2.4^b^	75.6 ± 4.6^b^	73.0 ± 3.3^b^	62.8 ± 4.3^b^	Y=−0.1134x+94.768	0.92
*E*. *globulus* EO	S	80.2 ± 1.3^b^	72.1 ± 3.1^b^	68.2 ± 1.9^b^	64.4 ± 4.1^b^	61.6 ± 2.7^b^	y= −0.1614x+89.732	0.89
	E	71.6 ± 3.4^c^	63.9 ± 2.3^c^	62.8 ± 2.4^c^	59.2 ± 1.1^c^	57.2 ± 2.3^c^	Y=−0.1829x+86.831	0.87
*I*. *verum* EO	S	82.6 ± 4.4^b^	79.0 ± 4.2^b^	76.4 ± 3.7^b^	74.2 ± 2.1^b^	66.0 ± 3.7^b^	y= −0.0973x+84.119	0.81
	E	74.4 ± 4.6^c^	69.6 ± 3.3^c^	65.8 ± 2.1^c^	62.0 ± 2.4^c^	55.3 ± 4.5^c^	y= −0.1193x+83.802	0.84
*Z*. *limonella* EO	S	87.2 ± 3.7^b^	80.6 ± 2.6^b^	77.6 ± 1.8^b^	72.4 ± 2.2^b^	60.8 ± 5.1^c^	Y=−0.1795x+84.648	0.85
	E	83.8 ± 3.8^b^	75.6 ± 3.4^b^	69.6 ± 1.5^c^	59.8 ± 4.1^c^	47.4 ± 6.1^d^	Y=0.1853x+80.167	0.81
Cypermethrin	S	73.4 ± 3.1^c^	64.2 ± 4.1^c^	63.0 ± 2.4^c^	56.6 ± 2.9^d^	48.8 ± 5.9^d^	y= −0.2017x+88.431	0.89
	E	69.6 ± 3.3^c^	63.5 ± 2.2^c^	62.4 ± 2.2^c^	56.0 ± 2.8^d^	43.6 ± 5.2^d^	y= −0.2306x+90.715	0.92
*F-test, Df*_*total*_		^*^,119	^*^,119	^*^,119	^*^,119	^*^,119		

There was an inverse relationship between percentage repellent activity and time of exposure. As the exposure time increased, the repellent activity percentage decreased, as shown by the regression curve in Fig. 3. This kind of relationship existed for all tested EO formulations, both in soybean oil and in ethyl alcohol. For example, soybean oil solution of *C*. *verum* showed the highest percentage repellent activity of 100% after 1 h of exposure, while after 48 h of exposure, it showed a lower 83.4%. For another example, *C*. *citratus* soybean oil solution showed 98.0% repellent activity after 1 h of exposure, but 79.0% repellent activity after 48 h of exposure. Following the same trend, after 1 h of exposure, the ethyl alcohol solution of *C*. *verum* showed 84.8% repellent activity, while after 48 h of exposure, it showed a lower 62.8%. Similarly, an ethyl alcohol solution of *C*. *citratus* showed 88.2% repellent activity after 1 h of exposure, while it showed a lower 62.0% repellent activity after 48 h of exposure.

Both 10% cypermethrin solutions in soybean oil and in ethyl alcohol showed a lower percentage repellent activity against *P*. *americana* adults than every EO solution in both kinds of solvents, at any length of exposure time. Cypermethrin solution in soybean oil (73.4% repellent rate) was only slightly more potent than cypermethrin in ethyl alcohol (69.6% repellent rate) after 1 h of exposure.

The effective repellent index (ERI) against *P*. *americana* adults of each of 10% solutions of five plant EOs and cypermethrin in soybean oil and in ethyl alcohol are presented in Table 4. As indicated by the ERI, all EO formulations were more repellent against *P*. *americana* adults than 10% cypermethrin. The ERIs of all EO formulations were always higher than that of cypermethrin at any time of exposure. The highest ERI observed was 1.71, achieved by 10% *C*. *verum* EO in soybean oil after 48 h of exposure, signifying that this EO formulation was 1.7 times more potent than 10% cypermethrin. Other EO formulations showed an ERI in the range of 1.09–1.62.

**Table 4:** Effective repellent indices (ERI) against *Periplaneta americana* adults of 10% solutions of five plant essential oils (EOs) and in soybean oil and in ethyl alcohol. *S* Soybean oil formulation, *E* Ethyl alcohol formulation. ERI = % repellent of each EO formulation/% repellent of cypermethrin.

Treatment	Solvent	Effective repellent index (ERI) / Time (h)				
		1	6	12	24	48
*C*. *citratus* EO	S	1.33	1.46	1.47	1.63	1.62
	E	1.27	1.23	1.42	1.24	1.42
*C*. *verum* EO	S	1.36	1.42	1.41	1.56	1.71
	E	1.23	1.25	1.21	1.30	1.44
*E*. *globulus* EO	S	1.09	1.13	1.08	1.14	1.26
	E	1.03	1.01	1.01	1.14	1.31
*I*. *verum* EO	S	1.17	1.23	1.21	1.31	1.35
	E	1.07	1.09	1.05	1.11	1.23
*Z. limonella* EO	S	1.19	1.26	1.23	1.28	1.25
	E	1.20	1.18	1.12	1.07	1.09

As shown in Tables 2 and 5 and, the ovicidal inhibition rate exhibited by the combined formulation was considerably higher (99.3%) than that exhibited by 10% *I*. *verum* EO (91.2%). In the same vein, the repellent activity against *P*. *americana* adults after 48 h of exposure exhibited by the combined formulation of 5% *I*. *verum* EO + 5% *C*. *verum* EO (see Table 6) was higher (89.5%) than that exhibited by *C. verum* EO (83.4%) (Table 3). Moreover, in both ethyl alcohol and soybean oil solvents, the formulation of the 5% *I*. *verum* EO + 5% *C*. *verum* EO combination exhibited a range of inhibition rates, 91.9–99.3%, which was very close to the 100% inhibition rate of cypermethrin. The most significant result was that the IRI of the combined EO formulation in soybean oil was very close to one (i.e., 0.99), indicating that its potency was almost identical to that of cypermethrin. Finally, the combined formulation in soybean oil (99.3% ovicidal inhibition rate) exhibited a higher efficacy than the combined formulation in ethyl alcohol (91.9% inhibition rate).

**Table 5:** Inhibition rate against *Periplaneta americana* eggs at 30 days after treated with a combined formulation of 5% *I*. *verum* EO + 5% *C*. *verum* EO and 10% cypermethrin, both in soybean oil and in ethyl alcohol solvents. *IRI* Inhibition Rate Index, *S* Soybean oil solution, *E* Ethyl alcohol solution, ^*^Treatment and non-treatment are significantly different at *P* *<* 0.05.

Treatment	Solvent	Number of hatched eggs ± SD		Inhibition rate (%)	*P*-value	IRI
		Treated	Untreated			
*I*. *verum* EO + *C*. *verum* EO	S	0.8 ± 0.2^*^	122.7 ± 2.1	99.3	0.01	0.99
	E	10.3 ± 1.9^*^	127.8 ± 5.1	91.9	0.05	0.92
Cypermethrin	S	0^*^	148.4 ± 3.7	100	0.01	–
	E	0^*^	152.3 ± 6.8	100	0.01	–

**Table 6:** Repellent activities against *Periplaneta americana* adults of a combined formulation of 5% *I*. *verum* EO + 5% *C*. *verum* EO and 10% cypermethrin, both in soybean oil and ethyl alcohol solvents. Mean percentage repellent activity within the same column followed by different letters are significantly different at *P<*0.05 (ANOVA and Tukey’s post hoc test). *ERI* Effective repellent index, *S* Soybean oil formulation, *E* Ethyl alcohol formulation, *R*^*2*^Regression coefficient, ^*^Significantly different at *P* *<* 0.05.

Treatment	Solvent	% Repellent / Time (h)					Regression equation of time versus % repellent	*R*^2^	ERI at 48 h
		1	6	12	24	48			
*I*. *verum* EO + *C*. *verum* EO	S	100^a^	95.7 ± 3.1^a^	94.2 ± 4.2^a^	93.3 ± 3.4^a^	89.5 ± 3.1^a^	Y=−0.0898x+94.725	0.95	1.94
	E	90.3 ± 3.7^a^	87.2 ± 4.3^b^	80.7 ± 3.6^b^	78.2 ± 4.3^b^	65.7 ± 2.8^b^	Y=0.1853x+80.167	0.81	1.47
Cypermethrin	S	70.3 ± 3.4^b^	62.4 ± 3.7^c^	60.5 ± 2.7^c^	58.4 ± 2.1^c^	46.2 ± 3.3^c^	y= −0.2115x+89.337	0.87	–
	E	69.7 ± 3.1^b^	60.3 ± 3.1^c^	58.2 ± 2.9^c^	55.3 ± 3.8^c^	44.7 ± 4.2^c^	y= −0.2215x+88.863	0.89	–
*F-test, Df *_*total*_		^*^,39	^*^,39	^*^,39	^*^,39	^*^,39			

As shown in Tables 3 and 6 and, the percentage repellent activity exhibited by the combined formulation was considerably higher than that exhibited by 10% *C*. *verum* EO and 10% cypermethrin. The combined formulation of 5% *I*. *verum* EO + 5% *C*. *verum* EO exhibited the highest repellent activity of 89.5% at 48 h of exposure, while 10% cypermethrin showed 44.7% repellent activity. The most significant result of this study was that the ERI of this combined EO formulation in both soybean oil and ethyl alcohol solvent was in the range of 1.47–1.94, signifying that this combined formulation was 1.47–1.94 times more potent in repellent activity than 10% cypermethrin. Regarding the influence of solvent on efficacy, following the same trend of influence on ovicidal inhibition rate mentioned above, the repellent activity exhibited by the combined formulation in soybean oil (89.5%) was higher than the activity exhibited by the combined formulation in ethyl alcohol (65.7%).

## Discussion

Several factors affected the extraction yield of plant essential oil, such as agricultural practice, cultivation area, and extraction method^26^. The observed essential oil yields from the five plants in this study were checked and confirmed against the values reported in the literature, and mostly, they agreed very well. Namely, the essential oil yield of *C*. *citratus* observed in this study was 1.15% v/w, while papers by Soonwera and Sittichok^24^, Aungtikun et al.^25^, and Verma et al.^26^ reported the yield of *C*. *citratus* as 0.50–1.5% v/w; the observed *C*. *verum* EO yield was 1.10% v/w, well-matched with 1.01–1.14% v/w reported by Aungtikun and Soonwera^27^ and Li et al.^31^; the observed *E*. *globulus* EO yield was 1.21% v/w (water distillation extraction method), well-matched with 1.2–3.0% w/w (water distillation) reported by Sebei et al^32^; and 1.10% v/w (steam distillation) reported by Joshi et al.^28^ and Soonwera and Phasomkusolsil^29^. The observed *I*. *verum* EO yield was 3.23% v/w, slightly lower than the 4.0–4.5% v/w, (water distillation) reported by Gholivand et al.^33^ and the 4.0% v/w (water distillation) reported by Aungtikun et al.^25^, and the observed yield of *Z*. *limonella* EO was 9.27% v/w, slightly lower than the 9.63% v/w reported by Charoensup et al.^30^. Moreover, although local species of *C*. *citratus* in Thailand and in India have the same number of constituents, the percentages of the constituents in their chemical profile are different^34^. Therefore, those yield percentages were determined and checked against the values in recent literature. Many factors influence the percentage of a major constituent in an EO’s chemical profile, such as plant maturity, harvesting time, and good harvesting practice^25^. The observed GC-MS chemical profiles of every EO were well-matched with those reported in a previous study. Namely, the percentage of geranial, the major chemical constituent of *C*. *citratus* EO observed in this study, was 46.33%, while papers by Soonwera and Sittichok^24^, Aungtikun et al.^25^, and Chauhan et al.^34^ reported the percentage geranial at 42.40–49.69% of the EO chemical profile; in addition, the observed major constituent of *C*. *verum* EO was cinnamaldehyde at 75.23%, well matched with 73.21% of the chemical profile reported by Aungtikun and Soonwera ^27^ and with 74.49% reported by Li et al.^31^, and lower than 90.17% reported by Chansang et al.^35^; the observed major constituent of *E*. *globulus* EO was 1,8-cineole, at 45.82%, well-matched with 42.60–44.54% of the chemical profile reported by Soonwera and Sittichok^24^ and Cotchakaew and Soonwera^36^; the observed major constituent of *I*. *verum* EO was trans-anethole, at 92.24%, well-matched with 88.32–94.0% of the chemical profile reported by Aungtikun et al.^25^ and Junior et al.^37^; and the observed major constituent of *Z*. *limonella* EO was limonene, at 28.13%, slightly higher than 18.62% reported by Imphat and Woottisin^38^, and lower than 43.63% reported by Charoensup et al.^30^, and 57.94% reported by Wongkattiya et al.^39^.

Nevertheless, there were some slight discrepancies, such as the yield and percentage in the chemical profile of the major constituents of *I*. *verum* and *Z*. *limonella* EOs. These discrepancies were likely due to the usage of different plant parts for EO extraction, a difference in the extraction procedures, and variations in genotypic, phenotypic, and agroecological factors (e.g., plant age, growth phase in plant development, amount of sunlight and moisture, as well as the temperature at the farm’s geographical location^40^.

High toxicity of plant EO in soybean oil against insect pests has been reported in recent literature. A study on EO toxicity against head louse eggs by Soonwera et al.^41^ concluded that Zingiberaceae plant EOs in soybean oil exhibited high toxicity to the eggs of head louse (*Pediculus humanus capitis*; Pediculidae: Phthiraptera), with a full 100% inhibition rate. In this study, 10% *I*. *verum* EO and the combined EO formulation in soybean oil showed high egg inhibition rates of 96.9% and 99.3%, respectively, against *P*. *americana*. This result was expected before the experiment started because the outcomes of many studies on *I*. *verum* EO against eggs of insect pests other than *P*. *americana* have been reported in the literature. Specifically, Sinthusiri and Soonwera^42^ reported that 10% *I*. *verum* EO exhibited markedly high oviposition deterrent and ovicidal activities against female houseflies (*Musca domestica*; Muscidae: Diptera), while Matos et al.^43^ reported that *I*. *verum* EO exhibited a high inhibition rate against the eggs of cowpea weevil, *Callosobruchus maculatus* (Bruchidae: Coleoptera). In addition to a high repellent activity against the adults, 10% *C*. *verum* EO and the combined formulation in soybean oil also exhibited a high repellent activity against *P*. *americana* adults—100% and 83.4% and 100% and 89.5% repellent activity at 1 and 48 h after exposure, respectively. Many papers have reported a high repellent activity of *C*. *verum* EO against several insect pests such as *Plodia interpunctella* (Pyralidae: Lepidoptera), *Bemisia tabaci* (Aleyrodidae: Homoptera), *Ae*. *aegypti* (Culicidae: Diptera), *Sitophilus zeamais* (Curculionidae: Coleoptera)^44^, *Sitophilus oryzae* (Curculionidae: Coleoptera)^45^, and ant^46^.

Unlike a large number of papers on individual EOs against *P*. *americana* mentioned above, papers on combined EO formulations against *P*. *americana* are limited. An example is a paper by Zibaee and Khorram^16^ reporting that a combined formulation of *E*. *globulus* + *R*. *officinalis* EOs exhibited a stronger insecticidal effect against adult *P*. *americana* than that of each individual EO component. Aungtikun et al.^25^ reported that a combined formulation of 0.5% *I*. *verum* EO + 0.5% geranial showed a higher insecticidal effect against adult house flies (*M*. *domestica*) than each individual EO.

Most notably, all EO formulations in soybean oil exhibited higher ovicidal and repellent activities against *P*. *americana* eggs and adults than all EO formulations in ethyl alcohol, in full agreement with findings from Sittichok and Soonwera^47^ that eight herbal EOs in soybean oil showed a higher inhibition rate against *P*. *americana* eggs than the same EOs in ethyl alcohol. Findings on repellent activity against adult mosquitoes in studies by Soonwera and Phasomkusosil^48^ and Phasomkusosil and Soonwera^49^ showed the same trend: 10% EOs from *C*. *citratus*, *C*. *nardus*, *Cananda odorata*, *Ocimum basilicum*, and *Z*. *cassumunar* in soybean oil weremore repellent and feeding deterrent against adult females of *Anopheles minimus*, *Ae*. *aegypti*, and *Culex quinquefasciatus* than the same EOs in ethyl alcohol. In the same vein, in this study, *C*. *verum* EO and the combined EO formulation exerted their potency better in soybean oil than in ethyl alcohol.

The soybean oil solvent in this study was pure soybean oil, but the ethyl alcohol solvent was 70% (v/v) ethyl alcohol in water. Therefore, the reason that a soybean oil solution of an EO is more repellent than the same EO in ethyl alcohol solution is that soybean oil is more lipophilic than ethyl alcohol, and a lipophilic solvent retards evaporation of EOs^49^. In this study, *C*. *verum* EO and the combined EO formulation in soybean oil provided a longer, more persistent repellent activity against *P*. *americana* than the same EO or combined EOs in ethyl alcohol, and the higher activity was attributed to this reason. On the other hand, an EO in a hydrophilic solvent evaporated faster and hence shorter, less persistent repellent activity. This claim is supported by a paper by Phasomkusosil and Soonwera^49^: 10% *Citrus sinensis* EO in soybean oil was more repellent (30–60 min) against adult females of *Cx*. *quinquefasciatus*, *Ae*. *aegypti*, and *An*. *minimus* than the EO in ethyl alcohol (less than 1.0 min). Another paper by Phasomkusosil and Soonwera^49^ and Phukerd and Soowera^50^ reported that 10% *Z*. *cassumunar* EO in soybean oil was more repellent (70 min) against adult females of *Ae*. *aegypti* than the EO in ethyl alcohol (30.0 min).

On the other hand, even though the reason that *I*. *verum* EO and the combined EO formulation were more potent in ovicidal activity in soybean oil than in ethyl alcohol is also the higher lipophilicity of soybean oil, but the mechanisms of action were not the same. The mechanism responsible for the higher ovicidal activity is that a lipophilic solvent can carry an EO through insect cuticles more readily because insect cuticles are mainly lipophilic^41^, hence more EO passes into the insect, providing more ovicidal activity from a higher amount of EO in its body^41,51,52^. This claim is supported by a conclusion from Rajashekar and Shivanandappa^53^ and Jankowska et al.^54^ that embryo and egg mortality were strongly affected by EO’s high cuticle permeability. The outcomes of this experiment confirm the expectation that every EO in soybean oil was more effective than the same EO in ethyl alcohol.

Regarding the high ovicidal activity (96.9–99.3%) against *P*. *americana* eggs of 10% *I*. *verum* EO among all tested individual EO formulations, this high activity is likely to be provided by its main constituent, trans-anethole^25^. Strong potency of trans-anethole against mosquitoes and houseflies was reported by Aungtikun et al.^25^ and Pavela^55^. Its mechanism of action was reported in Jankowska et al.^54^ and Bosch-Serra et al.^56^: namely, trans-anethole inhibited the development of embryo by blocking cytochrome P450 detoxification enzymes, retarding cell growth and juvenile hormone production and weakening the immune system, leading to eventual embryo death.

In the same way, the high repellent activity (100% at 1 h) against *P*. *americana* adults of 10% *C*. *verum* EO is likely to be provided by its main constituent, cinnamaldehyde^47^. Cinnamaldehyde has been reported to strongly repel *P*. *americana* adults (75.23%)^47^. The repellent activity of cinnamaldehyde is strong because it causes serious damage to their respiratory system^47^, so they always try to avoid it. This conclusion is supported by a claim by Devi and Devi^57^ that the mechanisms of action of cinnamaldehyde against insect pests were inhibition of its respiratory system by enzyme inhibition, cell membrane alteration, and reduced cell membrane integrity, as well as reduced cell respiration. Because of these reasons, *C*. *verum* EO showing the lowest ovicidal efficacy (in Table 2) makes sense because it does not have a constituent that strongly inhibited the eggs of *P*. *americana* like trans-anethole that *I*. *verum* EO has, hence it provided a low inhibition rate against the eggs of *P*. *americana* and house fly, as reported by previous works of Sittichok and Soonwera^47^ and Sinthusiri and Soonwera^42^. In the same vein, the graph of repellent activity of *C*. *citratrus* EO in Fig. 3 looks similar to that of *C*. *verum* EO but not that of *I*. *verum* EO because of them have geranial and cinnamaldehyde as their major constituent, while *I*. *verum* EO do not.

The overall findings from this study indicate that the combined formulation of 5% *C*. *verum* EO + 5% *I*. *verum* EO would be a good current alternative to cypermethrin because, first, it is equally or more potent than cypermethrin in ovicidal and repellent activities and, second, they should be less harmful to humans and non-target organisms and not be as persistent as cypermethrin in the environment. Findings supporting the first reason were as follows. First, against *P*. *americana* adults, *C*. *verum* EO and the combined EO formulation were 1.71–1.97 times more potent than cypermethrin in repellent activity, while the ovicidal activity of cypermethrin was only slightly more potent than those of *I*. *verum* EO and the combined EO formulation. Moreover, these findings are supported well by findings from a previous study by Sinthusiri and Soonwera^42^ that 10% cypermethrin showed a 100% inhibition rate against house fly eggs, while 10% *I*. *verum* EO showed a 97.33% rate. Second, findings by Ichikawa^58^ and Sharma et al.^59^ support the safety reason for replacing cypermethrin with a plant essential oil: cypermethrin is seriously toxic to the nervous and immune systems of humans, especially pregnant women and children, while findings by Aungtikun and Soonwera^27^ and Patra et al.^60^ support the claim that EOs are quickly degraded in the environment. Moreover, its safety to humans has long been established in Southeast Asia because it has been widely used as folk medicine since ancient times, and today, it is an irreplaceable food ingredient on some Thai menus as well as an active ingredient in modern, scientifically tested medicine (Tamiflu, an anti-influenza drug).

To conclude, the findings in this study indicate that 10% EO of *I*. *verum* provided the highest ovicidal activity against *P*. *americana* eggs among the five tested EOs, while 10% *C*. *verum* provided the highest repellent activity against *P*. *americana* adults. Moreover, they suggest that a formulation of their combination (5% *I*. *verum* EO+ 5% *C*. *verum* EO) in soybean oil can replace cypermethrin as an equally potent but much safer alternative agent for controlling *P. americana* populations.

## Materials and methods

### Schematic diagram summarizing the experimental design

**Figure 1:**
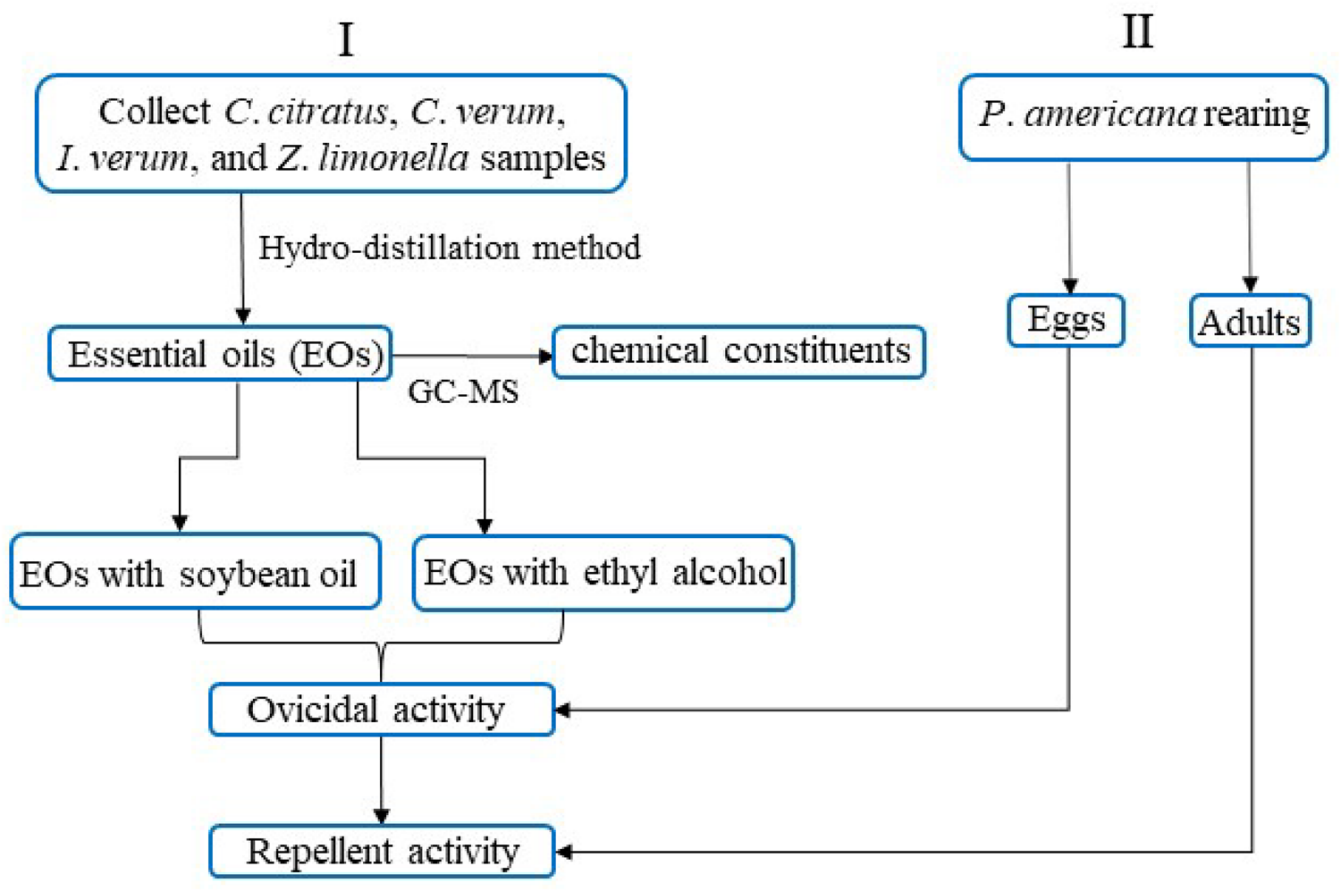
Schematic diagram of the experimental design.

Schematic overview of the experimental design of this current study is shown in Fig. 1.

### Plant materials

**Figure 2:**
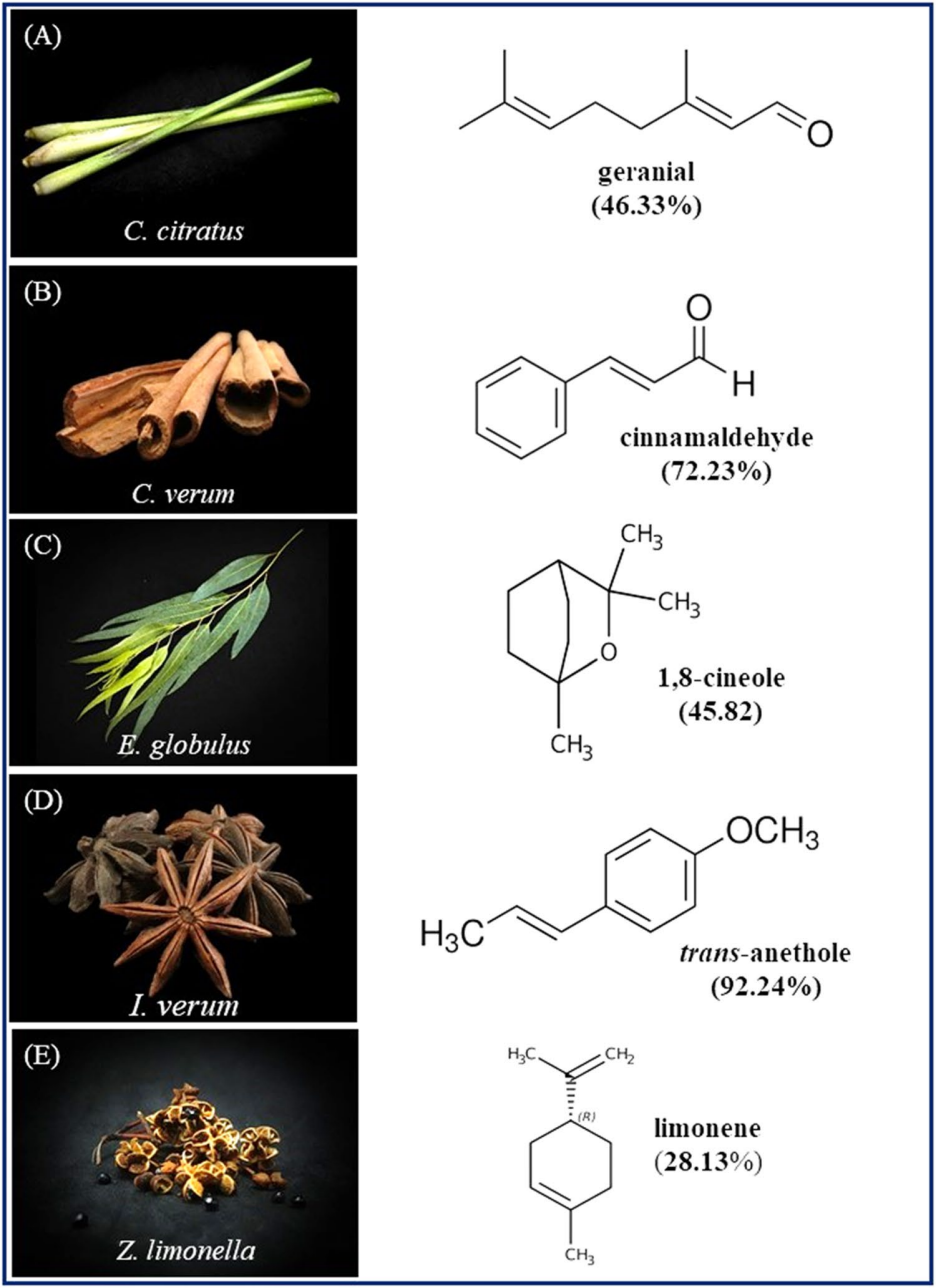
*Cymbopogon citratus* stems (**A**), *Cinnamomum verum* bark (**B**), *Eucalyptus globulus* leaves (**C**), *Illicium verum* fruits (**D**), and *Zanthoxylum limonella* fruits (**E**) as well as the chemical structures of their major essential oil (EO) constituents.

This study investigated five EOs extracted from some of the following five plants: *C*. *citratus*, *C*. *verum*, *E*. *globulus*, *I*. *verum*, and *Z*. *limonella*. Fresh stems of *C*. *citratus* (KMITL-CC), and fresh leaves of *E*. *globulus* (KMITL-EG) were collected from an organic farm run by King Mongkut’s Institute of Technology Ladkrabang (KMITL), Bangkok, Thailand (13.7563º N latitude, 100.5018º E longitude) and permit number KDS 2021/002, permitted by Dean of Faculty of Agricultural Technology, KMITL (Asst. Prof. Dr. Thongchai Phutthongsiri). Dried fruits of *Z*. *limonella* (KMITL-ZL) were purchased from a local market in Nan Province, northern Thailand. Dried bark of *C*. *verum* (KMITL-CV) and dried fruits of *I*. *verum* (KMITL-IV) were purchased from a Chinese medicine shop in Bangkok. Scientific identification of the five plants was accomplished by Dr. Sirawut Sittichok and Miss Jirapon Aungtikun, the herbal specialist at the KMITL Herbarium. All voucher specimens were deposited at the KMITL Herbarium, Faculty of Agricultural Technology, KMITL for future reference. This study complied with relevant local (KMITL) and national (the National Research Council of Thailand: NRCT) guidelines and legislation of Thailand. Photographs of those parts of *C. citratus*, *C. verum*, *E. globulus*, *I. verum*, and *Z. limonella* are displayed in Fig. 2 and the names of their major chemical constituents are listed in Table 1.

### Extraction of plant essential oils

**Figure 3:**
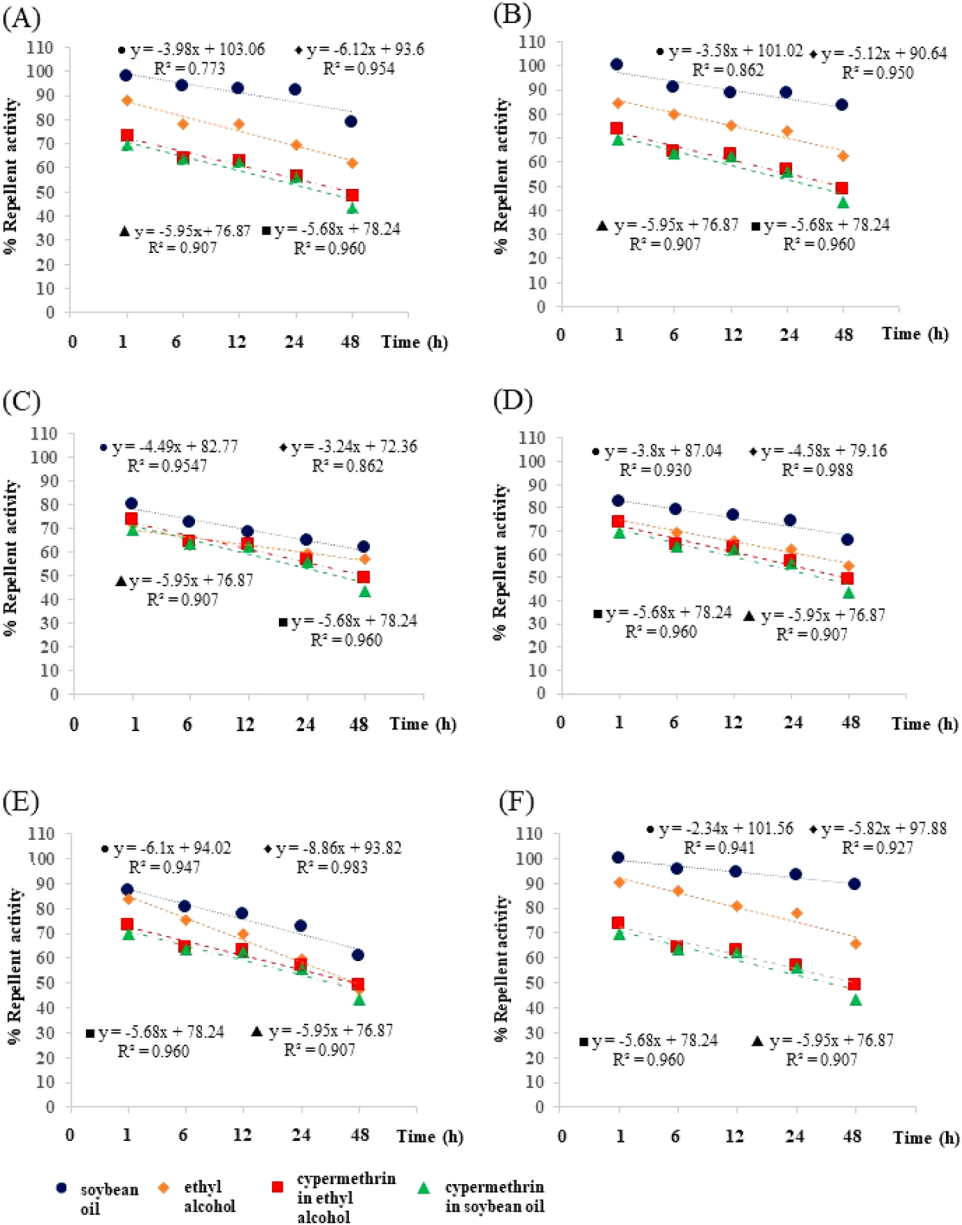
Relationships between percentage repellent activity and time (h) of five essential oils (EOs) and a combined formulation of two EOs in soybean oil and in ethyl alcohol against *Periplaneta americana*, compared to that of cypermethrin: *C*. *citratus* EO (**A**), *C*. *verum* EO (**B**), *E*. *globulus* EO (**C**), *I*. *verum* EO (**D**), *Z*. *limonella* EO (**E**), and 5% *I*. *verum* EO + 5% *C*. *verum* EO (**F**). Note: blue line represents the relationship between percentage repellent activity and time for each EO in soybean oil, orange line represents the same kind of relationships for each EO in ethyl alcohol, red line represents the relationship between percentage repellent activity and time for cypermethrin in soybean oil, and green line represents the same kind of relationship for cypermethrin in ethyl alcohol.

All tested plant EOs were extracted by the same hydro-distillation method^25,27^. In brief, 1,000 g of each plant part was cleaned, cut into small pieces, crushed, and extracted of its EO. Water to plant material ratio in the hydro-distillation process was 2:1 ratio, the rate of distillation was about two drops of EO per second. The extraction was completed in 5–6 h. Each EO was collected in 50 ml brown airtight vials and preserved at 4 °C for further bioassay and chemical constituent analysis. Each EO and a combined formulation of the EO with the highest ovicidal activity as well as the EO with the highest repellent activity were prepared into 10% solutions in soybean oil and in ethyl alcohol. The chosen concentration of these EOs and the combined formulation were already proven effective against adult *P*. *americana* and adult *M*. *domestica* in previous studies by Sittichok et al.^20^, Aungtikun et al.^25^, and Sittichok and Soonwera^47^. All formulations were stored under laboratory conditions (27 ± 2 °C and 70 ± 3% RH) until they were assayed (Fig. 3).

### Chemical constituent analysis

Chemical constituents of EOs from *C*. *citratus*, *C*. *verum*, *E*. *globulus*, *I*. *verum*, and *Z. limonella* were analyzed by gas chromatography-mass spectrometry (an Agilent Technology (USA) GC-MS system)^10,27^ at the Scientific Instrument Center of KMITL. A GC-MS instrument GC 6890-N (Agilent Technologies Co., Ltd. USA) was used, with an HP-5MS column (30 m × 0.25 mm i.d.) and a 0.25 μm film thickness. The 0.2 μl of samples of an ethyl alcohol solution of each EO were injected into the column. Helium (99.99%) was the mobile phase, flowing at a rate of 1 ml/min. The column oven temperature was first set at 50 °C and increased by 10 °C per min to reach 200 °C (held for 3 min), and then held at 270 °C. Every chemical constituent was identified with Agilent software (version G1701DA D.00.00). The identity of each identified chemical by GC-MS was then confirmed by a retention index comparison. The retention index (RI) of each ingredient was calculated with respect to the homologous series of *n-*alkanes (C_7_-C_30_). The obtained RI of each EO constituent was compared to the reference RI for that constituent, as reported in the chemical literature^61,62^.

### Source and purity of reagents

Standard *n-*alkanes (C_7_–C_30_) was procured from Sigma-Aldrich (USA). The positive control, 10% (w/v) cypermethrin (Detroy 10^®^), a common chemical insecticide was purchased from MD Industry Co., Ltd. (Pranakhonsri Ayutthaya, Thailand). The negative control, 70% (v/v) ethyl alcohol was purchased from T.S Interlab Limited Partnership, (Bangkok, Thailand) and pure soybean oil was purchased from Thai Vegetable Oil Co., Ltd. (Nonthaburi, Thailand). All chemicals/reagents employed in the study were of analytical grade.

### *P. americana* rearing

Twenty pairs of *P*. *americana* adults were obtained from the National Institute of Health, Department of Medical Sciences, Thailand’s Ministry of Public Health (13.85298°N latitude, 100.53008°E longitude). They were reared in an insectary laboratory, Faculty of Agricultural Technology, KMITL. The environmental conditions of the laboratory were a temperature of 27–28 °C and an RH of 68–70%. The *P*. *americana* adults were reared in black insectary boxes (ten pairs per 20.0 cm × 30.0 cm × 12.0 cm box) and fed 20 g of dog biscuit mixed with 10 g of powdered milk, and 10% glucose solution, supplemented with 5% multivitamin syrup solution soaked in sterile cotton sheets. After 180–240 days of rearing, they grew through stages of their life cycle, from eggs (ootheca) to nymphs and then to adults. Five-day-old ootheca and two-month-old adults were used in ovicidal and repellent bioassays, respectively.

### Ovicidal bioassay

The ovicidal activity provided by each EO formulation against *P*. *americana* eggs was determined by a topical application assay^42,47^. Two oothecae were placed in a 5-cm petri dish. Two dishes were placed in a black insectary box (16.0 cm wide × 26.0 cm long × 7.0 cm high): a treatment dish and a non-treatment dish. The treatment dish held eggs topically treated with 100 µL of each EO formulation or 10% (w/v) cypermethrin in soybean oil and in ethyl alcohol solvents, while the non-treatment dish held eggs topically pipetted with 100 µL of clean water. Each treatment was replicated five times. The number of hatched eggs was observed and recorded after 30 days of incubation (at 27–28 °C and 68–70% RH). The egg inhibition rate was calculated by the following formula^41^,

1 $${\text{Inhibition}}\;{\text{rate}}\;\left( \% \right) = \left[ {{\text{U}} - {\text{T}}/{\text{U}}} \right] \, \times \, 100,$$

where U is the total number of hatched eggs that were not treated with an active substance and T is the total number of hatched eggs treated with an EO solution or cypermethrin.

An inhibition rate index, as defined by Aungtikun and Soonwera^27^ as an index of efficacy comparison between an EO and cypermethrin, was calculated by the formula below,

2 $${\text{Inhibition}}\;{\text{Rate}}\;{\text{Index}}\;\left( {{\text{IRI}}} \right) = \frac{{\% \;{\text{Inhibition}}\;{\text{rate}}\;{\text{of}}\;{\text{each}}\;{\text{EO}}\;{\text{formulation}}}}{{\% \;{\text{Inhibition}}\;{\text{rate}}\;{\text{of}}\;{\text{cypermethrin}}}}$$

IRI <1 signifies that the EO formulation was less toxic than cypermethrin; IRI = 1 signifies that the EO formulation was equally toxic to cypermethrin; and IRI >1 signifies that the EO formulation was more toxic than cypermethrin.

### Repellent activity bioassay

The repellent activity of each EO formulation against *P*. *americana* adults was determined by a dual-choice application assay^47^. In the assay, each treatment formulation was dropped onto, absorbed by, and contained in half pieces of Whatman No.1^®^ filter paper (18.0 cm wide × 28.0 cm long). One piece was a non-treatment area for comparison with another piece of the treatment area. Two milliliters of clean water were dropped onto a non-treatment piece, and two milliliters of each treatment formulation were dropped onto a treatment piece. Both pieces were placed in a black insectary box (20.0 cm wide × 30.0 cm long × 12.0 cm high) in separate areas: treatment and non-treatment areas. Two plastic cups (5.0 cm in diameter × 4.0 cm high) containing food (2 g of dog biscuits) and drink (50 mL of 10% glucose solution) for the cockroaches were placed on top of each treatment and non-treatment filter paper piece. Five male adults and five female adults of *P*. *americana* were released at the center of the insectary box. Either in soybean oil or ethyl alcohol solvent, 10% w/v cypermethrin was used as a positive control for the corresponding treatments of every EO in soybean oil and in ethyl alcohol.

Each experiment was repeated ten times. The number of cockroaches situated in the non-treatment area was observed and recorded at 1, 6, 12, 24, and 48 h. The repellent rate was calculated by the following formula^18^:

3 $${\text{Repellent rate = }}\left[ {{\text{N}} - {\text{T}}/{\text{N}} + {\text{T}}} \right] \times 100,$$

where T is the number of *P*. *americana* situated in the treatment area at the time of observation, and N is the number of *P*. *americana* situated in the non-treatment area.

The effective repellent index was calculated by the following formula^18^:

4 $${\text{Effective}}\;{\text{repellent}}\;{\text{index}}\;\left( {{\text{ERI}}} \right) = \frac{{\% \;{\text{repellent}}\;{\text{of}}\;{\text{each}}\;{\text{EO}}\;{\text{formulation}}}}{{\% \;{\text{repellent}}\;{\text{of}}\;{\text{cypermethrin}}}}$$

ERI <1 signifies that the EO formulation was less repellent against *P. americana* than cypermethrin; ERI = 1 signifies that the EO formulation was equally repellent to cypermethrin; and ERI >1 signifies that the EO formulation was more repellent than cypermethrin.

### Statistical analysis

The numbers of hatched eggs and unhatched eggs in the treatment and non-treatment areas were analyzed for significant differences at *P*<0.05 by a paired *t-*test method*.* The repellent activity and ovicidal assays were completely randomized design. Significance differences at *P*<0.05 were determined by ANOVA (one-way analysis of variance) and Tukey’s post hoc test. The coefficients of the regression equation of time versus % repellent activity were determined by SPSS statistical software.

## Data Availability

All data generated or analyzed during this study are included in this published article [and its supplementary information files].

## Abbreviations

EO: essential oil
EOs: essential oils
IRI: Inhibition rate index
ERI: Effective repellent index
